# Synthetic consortia of nanobody‐coupled and formatted bacteria for prophylaxis and therapy interventions targeting microbiome dysbiosis‐associated diseases and co‐morbidities

**DOI:** 10.1111/1751-7915.13355

**Published:** 2018-12-21

**Authors:** Kenneth Timmis, James Kenneth Timmis, Harald Brüssow, Luis Ángel Fernández

**Affiliations:** ^1^ Institute of Microbiology Technical University Braunschweig Braunschweig Germany; ^2^ Athena Institute Vrije Universiteit Amsterdam Amsterdam The Netherlands; ^3^ Division of Animal and Human Health Engineering Department of Biosystems Katholieke Universiteit Leuven Leuven Belgium; ^4^ Department of Microbial Biotechnology Centro Nacional de Biotecnología Consejo Superior de Investigaciones Científicas Madrid Spain

## Abstract

Designed nanobody‐linked synthetic consortia for microbiota dysbiosis therapies. A. Nanobodies (Nb) are selected for specific antigens on target bacteria destined for a synthetic therapy consortium that may consist of two (B) or multiple (C) members. For the treatment of dysbiosis co‐morbidities requiring two functionally distinct consortia, these may be linked through a common member to generate a single bi‐functional microbiota therapy (D).

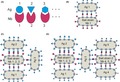

## Microbiome dysbiosis‐associated medical conditions and natural microbiota as therapeutic agents

Microbiome dysbiosis (Brüssow, 2016) – the deviation from a desired, equilibrated, health‐associated microbiota^1^ – especially of the major microbial organ, the gastrointestinal tract, is being increasingly associated with diverse medical conditions. The classic disease resulting from dysbiosis is antibiotic therapy‐induced pseudomembranous colitis, caused by *Clostridium difficile*. More recently, allergies and other, often severe, conditions, such as inflammatory bowel disease, cancer, psychological disorders and various cardiometabolic diseases such as diabetes mellitus and obesity, have been associated with microbiota dysbiosis. Cardiometabolic diseases are of particular importance, as they significantly increase the risk of developing or exacerbating incident cardiovascular disease – the leading cause of death across the OECD world (circa 36%) and associated with 17% (on average) of total healthcare costs (OECD, 2015, 2017). The list of dysbiosis‐disease associations continues to grow. Causal relationships have, for the most part, not yet been established, and we are still far from understanding pertinent disease aetiology (Brüssow, 2016).

A central goal of dysbiosis therapy is to provide an ecological impetus for re‐establishment of the microbiota ecophysiological diversity and functional coherence needed for ‘healthy’ performance of the relevant surface/organ/metabolic network. A simple therapy option is to supplement the dysbiotic microbiota with a sample of corresponding microbiota from a healthy donor, i.e. faecal microbiota transplantation, a therapy that has achieved excellent outcomes in the case of *C. difficile*‐caused colitis (Eiseman *et al*., 1958; Seal *et al*., 1989; Tvede and Rask‐Madsen, 1989; van Nood *et al*., 2013; Rossen *et al*., 2015). As a consequence of this success, a range of new therapeutic interventions for correcting dysbiosis are being explored, which involve the administration of selected live microbes, microbial mixtures and natural microbial consortia, as well as pre‐ and probiotics, for both therapy and prophylaxis. However, since it has not yet been established which component(s) of the faecal sample (entire faecal material, spores – e.g. of clostridia, the cell‐free fraction, the combinations of certain specific faecal microbes, etc.) in faecal microbiota transplantation determine(s) its effectiveness, the potential of novel microbiota therapies must, for the moment, be viewed with both optimism and caution.

## Challenges facing natural microbiota as therapeutic agents

Important issues related to the use of natural consortia, such as faecal samples, as therapies for microbiome dysbiosis are *inter alia* safety, standardization, reproducibility and quality control. Faeces from apparently healthy donors of course contain (low numbers of) pathogens, which are normal members of the natural flora, and ordinarily do not cause disease in otherwise healthy individuals. Sometimes, however, they may contain infectious agents that qualitatively (e.g. hepatitis virus, HIV, *Campylobacter*,* Helicobacter*, etc.) or quantitatively pose a significant risk for patient safety. Moreover, there exists a high level of diversity and variability in microbiota compositions among individuals, and within individuals over time, so achieving standardization of donor samples is a major challenge (Costello *et al*., 2009; Rajilic‐Stojanovic *et al*., 2012). Perhaps of even more concern is the fact that both dysbiosis and potential therapies currently constitute black boxes, with little or no understanding of underlying processes, regulatory controls and switches, and causal relationships. Improving our understanding of the physiological processes underlying dysbiosis:disease relationships is crucial not only for developing new interventions, but also for establishing biological and associated regulatory standards and guidance to facilitate timely approval and implementation of novel biomedical interventions.

## Synthetic microbiota for therapies

The trend will thus inexorably be towards the development of single, extremely well‐characterized, pure and fermenter‐propagated microbial strains, for those conditions for which single strains prove to be effective therapeutics. This is not only because of simplicity, safety, standardization, quality control and regulatory issues, but also because of the imperative of further development for improved effectiveness, predictability, etc., and the pertinent potential that powerful synthetic microbiology design approaches offer. And, most importantly, because single strain therapies will greatly simplify the experimental investigation of underlying ecophysiological processes and establishment of causalities.

However, since dysbiosis involves complex compositional, and especially functional, ecophysiological changes in the microbiota, restoration of a healthy microbiota will often require the administration of multiple, functionally diverse strains delivered in the form of a coherent community. While one approach may be to simplify a natural community, such as one derived from a faecal sample – for example by propagation in an artificial gut bioreactor – its relatively poorly characterized members may still pose risks and issues of reliability and reproducibility. Alternatively, synthetic consortia assembled from well characterized individual strains, propagated as pure cultures, may be developed that exhibit low risk profiles, and properties that are standardized, predictable and effective.

## Design of physically‐linked microbial consortia by means of nanobody surface display

The main challenge facing the use of synthetic consortia is their lack of intrinsic physical and functional cohesion, and their uncertain ecological fate *in situ*. In particular the potential for rapid loss of one or more members, due for example to poor competitivity in dynamic environments like the GI tract, and the possible ensuing loss of beneficial functionalities of the administered consortium, can impede intervention effectiveness.

**Figure 1:**
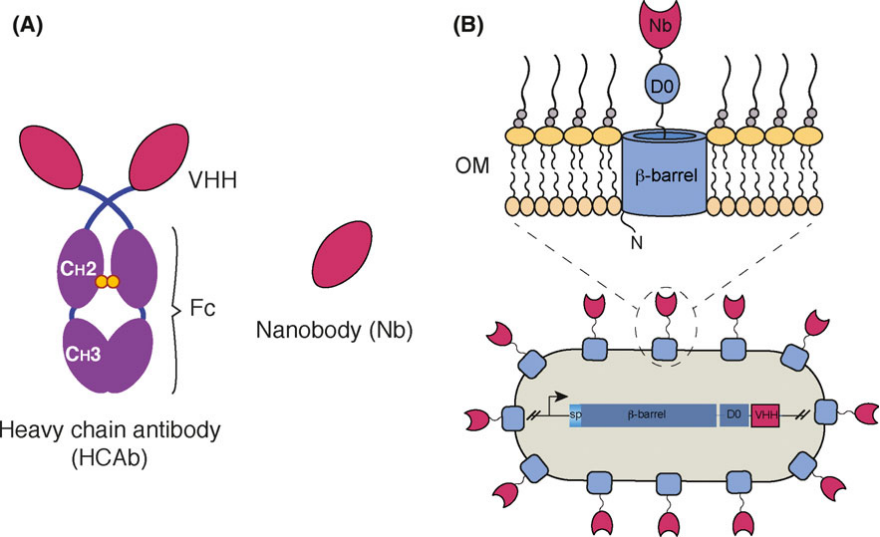
Nanobody display and synthetic adhesins of *Escherichia coli*. A. Structure of a camelid heavy chain‐only antibody (HCAb), indicating the constant Fc region and variable VHH domains. Nanobodies (Nbs) are the antigen‐binding VHH domains produced by engineered microbes. B. Surface display of Nbs on the *E. coli* cell surface, through their fusion to intimin N‐fragment 1‐654 comprising ß‐barrel and Ig‐like D0 domain, which anchors the Nb to the bacterial outer membrane (OM).

To counteract a possible lack of coherence of a synthetic consortium when propagated in a natural setting, de Lorenzo proposed in 2008 the use of surface displayed recombinant antibodies – nanobodies (Nbs) – to physically link the consortium partners (de Lorenzo, 2008; see also Veiga *et al*., 2003). Nbs are recombinant single domain antibodies derived from the variable regions of a special class of immunoglobulins lacking light chains that are naturally present in members of the camelidae (e.g. camels, dromedaries, llamas, alpacas, etc.; (Muyldermans, 2013). The variable regions of heavy chain‐only antibodies (HCAbs), referred to as VHH in Fig. 1A, have multiple adaptations that endow them with solubility and the ability to bind antigens (Ags) in the absence of a paired light chain. The small size (ca. 14 kDa) and superior biophysical and antigen‐binding properties of Nbs make them ideal candidates for diverse applications requiring the selective recognition and binding to Ags of different entities, including viruses, bacteria, and eukaryotic cell targets (Oliveira *et al*., 2013; Chakravarty *et al*., 2014; Steeland *et al*., 2016; Van Audenhove and Gettemans, 2016). Importantly, high affinity Nbs of desired specificity can be selected from libraries of VHH gene segments, amplified from peripheral blood lymphocytes of immunized animals, and cloned and expressed in bacteriophages, *E. coli* or yeast cells (Salema and Fernandez, 2017).

For the purpose of displaying Nbs on the bacterial surface, they are fused to an N‐fragment of intimin (residues 1–654), comprising its ß‐barrel and first extracellular Ig‐like domain (D0) (Fig. 1B), which stably anchors them in the outer membrane of Gram negative bacteria. The intimin anchor is highly stable *vis‐à‐vis* strong denaturants (e.g. SDS, urea) and proteases (e.g. trypsin) (Bodelón *et al*., 2009), allows for high density display of Nbs on *E. coli* surfaces, and thereby the efficient selection of binders from VHH libraries (Salema *et al*., 2013). Importantly, intimin‐Nb fusions have been shown to constitute robust synthetic adhesins for *E. coli*, that mediate their rapid and specific bonding to cognate antigens on target cells (Piñero‐Lambea *et al*., 2015a).

**Figure 2:**
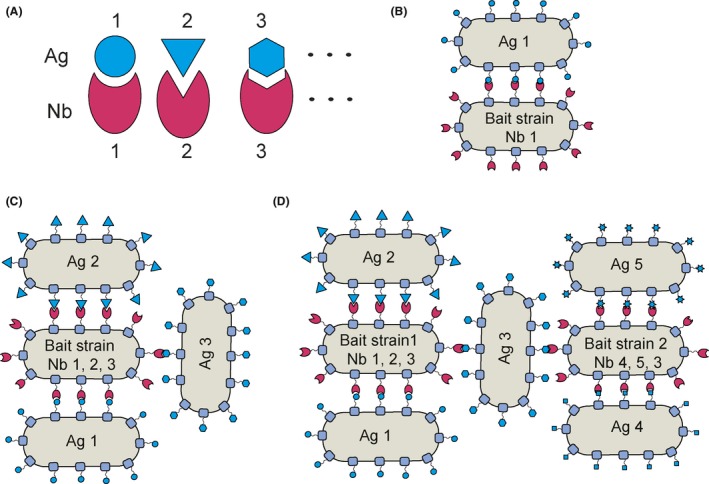
Designed nanobody‐linked synthetic consortia for microbiota dysbiosis therapies. A. Nanobodies (Nb) are selected for specific antigens on target bacteria destined for a synthetic therapy consortium that may consist of two (B) or multiple (C) members. For the treatment of dysbiosis co‐morbidities requiring two functionally distinct consortia, these may be linked through a common member to generate a single bi‐functional microbiota therapy (D).

Recent progress has shown that Nbs can be used for the controlled formation of combinations of different *E. coli* derivatives (Glass and Riedel‐Kruse, 2018) in predefined architectures, and provided proof of principle of the design of synthetic consortia based on what might be called a ‘bait’ strain displaying Nbs, that stably bind to one or multiple target strains bearing cognate Ags, either natural or engineered, expressed on their surfaces (Fig. 2A–C). For microbiota therapies, either the bait strain or one of the target strains should be a good colonizer of the target organ in order that the synthetic consortium persists *in situ* for the period needed for attainment of the therapeutic effect.

By enabling the *à la carte* modular design and generation of synthetic, physically‐linked consortia of well‐characterized microbes, the nanobody surface display platform assures the functional coherence needed for synthetic microbiology‐based precision medicine interventions, and thus opens up the field for novel approaches to treating microbiota dysbiosis. The challenge is to identify, characterize and demonstrate efficacy and, subsequently, effectiveness, of strains appropriate for this purpose (for a light‐hearted treatment of some of the relevant issues, see Timmis *et al*., 2019, this issue).

## Further potential applications of nanobody surface display in live microbe therapies

The nanobody surface display platform also enables the specific targeting of individual microbes, and designed consortia, to other relevant biological surfaces of interest, for example, those of epithelial cells, to prolong residence time and hence therapy duration, or of the gut‐associated lymphoid tissue (e.g. Peyer's Patches), to achieve immunomodulatory goals, such as stimulation of mucosal immunity against specifically‐presented antigens, or localized secretion of anti‐inflammatory cytokines or antibodies produced by one or more of the delivered bacteria (Piñero‐Lambea *et al*., 2015b; Bermudez‐Humaran and Langella, 2018).

**Figure 3:**
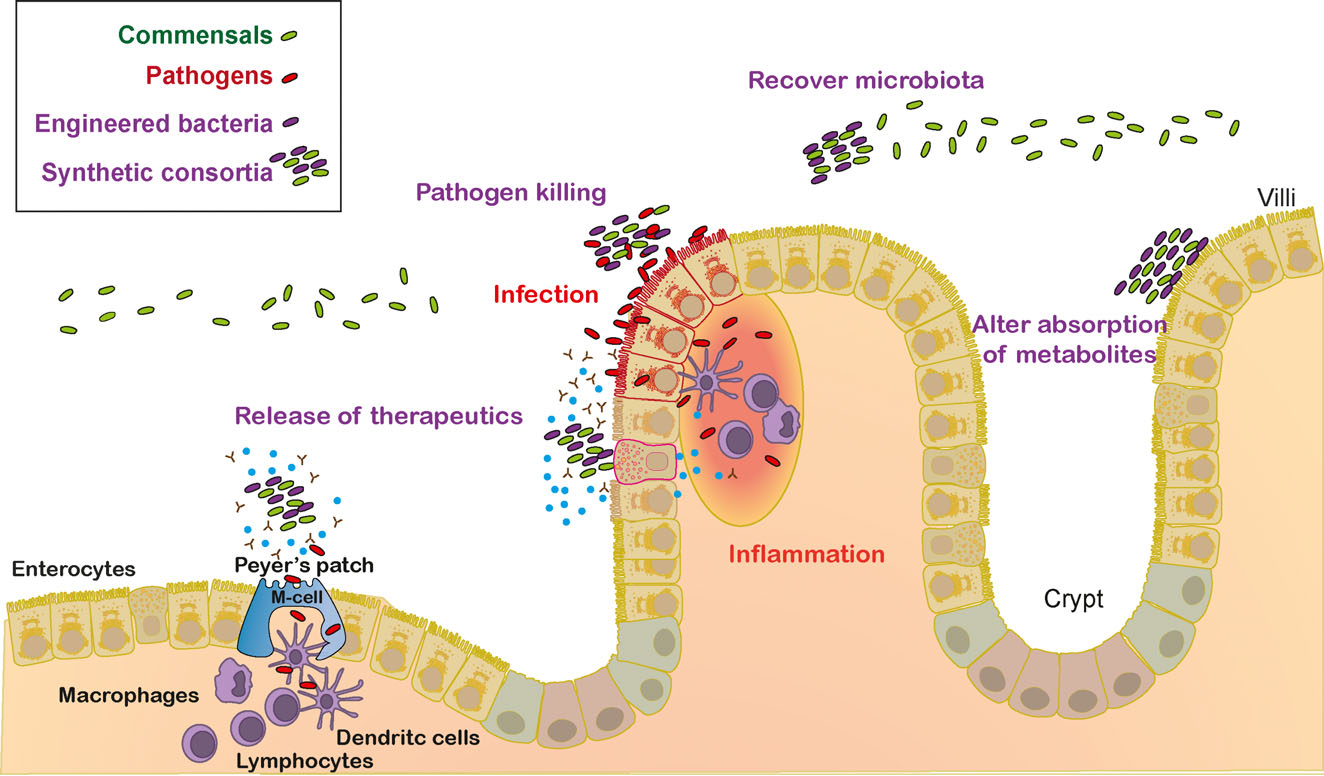
Further potential therapeutic applications of synthetic consortia linked by surface‐displayed nanobodies. The figure portrays various interactions of synthetic consortia and the host microbiota and/or niches and tissue sites of the gastrointestinal tract mediating therapeutic activities. For instance, engineered bacterial strains in the consortia may deliver antigens or release biotherapeutics (e.g. antibodies, cytokines) locally to promote immune responses, e.g. in the Peyer's Patches, or reduce mucosal inflammation. Bacteria in the synthetic consortia may also display nanobodies to “trap” pathogens and kill them specifically by delivery of antibacterial toxins (e.g. microcins, effectors of type 6 secretion systems). These activities may favour colonization of the gastrointestinal tract by beneficial commensals during and after disease and dysbiosis. In addition, nanobody‐driven attachment of synthetic consortia to the mucosal surface may enhance therapeutic actions either favouring or preventing the absorption of specific metabolites by the host.

Creating individual microbes, or combinations thereof, that target and bind to GI tract mucosal epithelial cells and produce beneficial metabolites may enhance absorption of such metabolites by the gut (Fig. 3) (Chen *et al*., 2014). Alternatively, consortia may be designed both to eliminate toxic metabolites, such as phenylalanine in patients with phenylketonuria (Isabella *et al*., 2018), and to attach to the host mucosal surface, to create a bioactive barrier that reduces the uptake of such metabolites (Fig. 3). Another potential application of particular interest in relation to the current antibiotic resistance threat is to combine expression of Nbs specific for surface localized Ags of pathogenic bacteria, and antibacterial products, such as microcins (Hwang *et al*., 2017) or toxins delivered by type 6 secretion systems (Alcoforado Diniz *et al*., 2015; Chassaing and Cascales, 2018), in order to target and kill pathogens, and hence reduce disease severity and duration. A particularly interesting potential application of Nb engineered bacteria is in tumour imaging and therapy using bacteria exhibiting tropisms for tumours (Felgner *et al*., 2017): Piñero‐Lambea *et al*. (2015a) report that non‐pathogenic *E. coli* bacteria displaying synthetic adhesins against tumour cells effectively colonize solid tumours at doses two‐orders of magnitude lower than those needed with control bacteria, suggesting a means of reducing the risks of this type of bacterial therapy (Chien *et al*., 2017). Such potential live therapeutic interventions (see e.g. Alvarez and Fernandez, 2017), and others, now acquire a new developmental potential, through the possibility of Nb‐mediated addition of one or more microbial partners able to contribute further beneficial functionalities.

### Multimorbidity and potential applications of synthetic microbiota in precision prophylaxes and therapies

One major, and arguably the most alarming, trend in the healthcare domain is multimorbidity. For example, according to a recent (2018) review for the UK (Tran *et al*., 2018), the number of individuals with incident cardiovascular disease and at least 5 co‐morbidities increased fourfold between 2000 and 2014, with 60% of co‐morbidities being of cardiometabolic nature (the most serious of which are chronic kidney disease, diabetes mellitus, hyperlipidaemia, hypertension, obesity and peripheral arterial disease). Based on current trends for diabetes mellitus, hypertension and obesity incidence, and tobacco consumption, cardiovascular mortality rates for individuals below 70 years of age are projected to increase by more than 30% globally over the next decade, to ca. 8 million deaths per annum (Antman and Loscalzo, 2016). Importantly, disease:disease interactions and polypharmacy (that often leads to serious adverse drug reactions caused by conflicting medications (see e.g. Bouvy *et al*., 2015)), frequently exacerbate health deterioration – the mechanisms of which mostly remain poorly understood at the molecular level – and limit treatment options for multimorbid patients (see e.g. Ording and Sorensen, 2013; Battegay *et al*., 2017;).^2^

The example of cardiovascular and cardiometabolic disease(s) is particularly relevant to the issue of microbiome therapies because both have been associated with microbiome dysbiosis, and hence may in some instances represent dysbiosis‐associated morbidity clusters. Moreover, as further microbiome dysbiosis‐associated diseases are discovered, and our understanding of the ecophysiological‐metabolic‐regulatory processes underlying dysbiosis and disease advances, it is to be expected that multiple distinct root causes of specific microbiota dysbiosis phenotypes and associated specific disease symptoms will be identified, that, in turn, will increasingly require disease reclassification based on cause. For example, a condition like obesity may well be a clinical phenotype that will subsequently be revealed to encompass several distinct diseases (leading e.g. to the identification of type 1, 2, 3, etc., obesity). The existence of dysbiosis‐associated morbidity clusters, some of which will necessitate two or more, perhaps confounding, therapeutic consortia having distinct therapeutic aims is therefore not unlikely.^3^ However, although multimorbidity will significantly increase complications of therapeutic material preparation, treatment and monitoring, the nanobody surface display platform, which is in principle upscalable in terms of the number of members of the synthetic consortium, should allow the construction of bi/multifunctional, non‐confounding consortia (Fig. 2D) that have the same preparation, treatment and monitoring requirements as monofunctional consortia.

As stated above, it is to be expected that there will be considerable variation in the causes of microbiota dysbiosis, as well as individual‐specific variation in disease manifestation, responses and therapy effectiveness profiles, even for patients with similar symptoms. This host‐specific variation is the basis of precision/personalized medicine – in its current form, the design of treatments for specific patient cohorts but, in future, increasingly the tailoring of interventions to individual patients (Jameson and Longo, 2015). Future precision medicine will undoubtedly include the personalization of live microbial communities for the treatment of microbiota dysbioses. A growing toolbox of well‐characterized and ‐tested strains exhibiting known therapeutic functionalities, together with the Nb surface display platform for the creation of *à la carte* consortium combinations, will not only provide the means of creation of individual therapies but also enable advancement of our understanding of the basis of such host variations.

## The wider context: systems medicine and translation

As alluded to above, our current understanding of causal relationships between microbiome dysbiosis and the aetiology of a variety of diseases, and clusters thereof, is in its infancy. However, recent advances in genome mapping and genome‐wide association studies and next generation sequencing, increases in the capacity and sophistication of big‐data collection and analytics (e.g. machine learning), utilization of electronic health records, etc., and the orientation towards a biomolecular network‐based, multidisciplinary and holistic *systems medicine* approach – the application of the principles of systems biology to the modern healthcare field (Hood *et al*., 2004, 2012) – have revolutionized biomedical research and development. Systems medicine is significantly advancing our understanding of, and thereby redefining, what constitutes health and disease (in relative terms), and which perturbations in protein and gene regulatory networks cause or predict a shift in the health:disease continuum (Sagner *et al*., 2017). This approach has already facilitated significant advances in health technologies, such as biomarker based liquid biopsy molecular diagnostics (Noell *et al*., 2018), and will in the future even more rapidly transform methods and techniques to investigate and analyse complex data‐sets, develop new diagnostics, prophylaxes and therapeutics to address complex and pressing healthcare needs with modern and stratified clinical interventions (Wolkenhauer *et al*., 2013; Bjornson *et al*., 2016; Apweiler *et al*., 2018; Noell *et al*., 2018). Although pervasively important for developing novel pharmacogenomic precision medicine interventions, such as recently introduced antibody‐based immunotherapies for leukaemia (see e.g. Maude *et al*., 2018), the systems medicine approach lends itself perfectly to the exploration and development of novel synthetic, scalable and modular microbial consortia‐based precision medicine interventions that are being discussed here.

Central to maximising both the leverage of the systems medicine approach for research and development of microbiome dysbiosis‐based novel interventions, and their effective and timely translation into clinical practice – will be *inter alia*

i the institution of population‐wide periodic microbiome sampling and analyses, i.e. data collection and interrogation (see e.g. Timmis and Timmis, 2017),
ii a shift towards more appropriate financing mechanisms (e.g. combined push‐ and pull‐incentives) to streamline translation and trialling of promising novel interventions (Renwick and Mossialos, 2018), and
iii utilization of knowledge valorization models to reduce information asymmetries between stakeholders and, in turn, improve the prospects of commercialising relevant new knowledge generated in public and private research and development organizations (Van de Burgwal *et al*., 2018).

## Conclusions

In summary, gazing into our Crystal Ball, we see the nanobody surface display approach at the centre of prevention and therapy measures for microbiota dysbiosis‐associated diseases, and indeed for other live microbe‐based interventions, in providing the glue of a technology platform (see e.g. Fraile *et al*., 2013) for the *à la carte* assembly of precise, modular, stable, synthetic consortia with designed functionalities for systems medicine‐based precision interventions. A growing toolbox of well‐characterized consortium members of proven utility will form the modules for consortium assembly. Such highly characterized modules and simple consortia will facilitate the development of improved, second generation microbial therapies, especially through the application of synthetic microbiology approaches. Most importantly, they will enable experimental investigation of underlying ecophysiological and metabolic processes and causalities of complex host:microbiota:symptom interactions that are currently black boxes, and thereby ultimately lead to a fundamental understanding of both physiology and dysbiosis of our microbiome surfaces, especially of the GI tract, and diseases channelled via the various gut‐organ axes.

## Notes

1 An *equilibrated, health‐associated microbiota* is currently not well defined and there exist substantial ethnic variations, rendering generalizations and morbidity associations difficult.

2 A major problem associated with multimorbidity is the fact that, although over the past two decades multimorbidity incidence, or rather the recognition thereof, and overall expenditures for multimorbidity management have increased at an accelerating rate, evidence of efficacy, effectiveness and efficiency of conventional (one‐size‐fits‐all) treatments, let alone of precision (personalized) medicine interventions, for multimorbidity is scarce and clinical guidelines are virtually non‐existent. One important reason is that conventional randomised clinical trials, which are considered the gold standard trial design, usually test one diagnostic and/or intervention targeting one specific condition/genotype and tend to explicitly exclude multi‐morbid patients in order to reduce the risk of bias and confounders, and to control for externalities (Ording and Sorensen, 2013; Battegay *et al*., 2017; Tran *et al*., 2018).

3 A further complication would be co‐morbidities involving both a classical disease, like cancer, and a microbiota dysbiosis‐associated disease, where the traditional therapy may affect the microbiota therapy.
